# Comparative biomolecular analysis of normal and healed skin tissue from diabetic and non-diabetic rats using Raman spectroscopy

**DOI:** 10.1007/s10103-026-04961-x

**Published:** 2026-07-25

**Authors:** Willy Leite Lima, Giovanna Garcia Vieira, Luciano Gonçalves da Nóbrega, Larissa Emily de Almeida Maciel, Bruno Araújo Serra Pinto, Landulfo Silveira Jr., Thaís Porto Amadeu, Nilton Maciel Mangueira

**Affiliations:** 1 Post-Graduate Program in Medical Sciences, Rio de Janeiro State University – UERJ, RJ Rio de Janeiro, Brazil; 2 Special Coordination of Morphology, Federal University of Maranhão – UFMA, MA São Luís, Brazil; 3 Anhembi Morumbi University – UAM, SP São Paulo, Brazil; 4 Center for Innovation, Technology and Education – CITÉ, SP São José dos Campos, Brazil; 5 School of Medicine, University Center of Maranhão – CEUMA, MA São Luís, Brazil

**Keywords:** Rat skin tissue, Diabetes, Healing, Biochemical composition, Characterization, Raman spectroscopy, Principal component analysis

## Abstract

Diabetes mellitus (DM) is a metabolic disorder characterized by chronic hyperglycemia that disrupts skin repair through alterations in tissue biochemical composition and structural organization. This study compared Raman spectral profiles of normal and healed skin tissue in non-diabetic (Non-Db) and diabetic (Db) Wistar rats, based on the hypothesis that diabetes alters skin molecular composition during healing. Twelve Wistar rats were divided into Non-Db and Db groups (*n* = 6 per group). DM was induced by of alloxan monohydrate injection (150 mg/kg). Dorsal skin excision was performed under anesthesia on day 0 (normal tissue) and day 14 (healed tissue), resulting in 24 samples. Raman spectra were acquired (830 nm excitation, 400–1800 cm^–1^ range, ~ 4 cm^–1^ resolution, 30 s integration time) in sextuplicate per sample. Data were analyzed by principal component analysis (PCA), followed by ANOVA or Kruskal-Wallis tests. The Raman spectra mainly characterized by features attributed to structural proteins and lipids from epidermal and dermal layers, including collagen, elastin, keratin, and amino acids, fatty acids, ceramides, phospholipids and cholesterol. PCA revealed group-dependent variations in protein- and lipid-associated bands. Increased in protein related features accompanied by a relative reduction in lipids contributions suggested disruption of protein-lipid balance during extracellular matrix remodeling, suggesting molecular alterations in healed skin tissue associated with DM. Raman spectroscopy showed changes in the biomolecular profile of skin healing in diabetic tissue compared with non-diabetic, evidencing a distinct repair pattern marked by modified collagen related features and enhanced membrane lipid signatures in healed diabetic skin.

**Supplementary Information:**

The online version contains supplementary material available at 10.1007/s10103-026-04961-x.

## Introduction

Diabetes mellitus (DM) is a chronic metabolic disorder characterized by sustained hyperglycemia resulting from insufficient insulin secretion and/or reduced insulin responsiveness [1, 2]. It is among the fastest-growing global health problems, currently affecting over 537 million people worldwide, with projections indicating that the number will reach 783 million cases by 2045, mainly driven by demographic and lifestyle factors such as population aging, physical inactivity, and high intake of sugars and refined carbohydrates [1, 2]. Beyond metabolic dysregulation, DM compromise’s immune function, vascular integrity, and tissue regeneration, particularly in the skin, leading to delayed wound healing, chronic ulcers, and increased risk of infection and amputation [3, 4].

Skin tissue acts as a multifunctional interface between the body and the external environment, providing protective, sensory, immunological, and thermoregulatory functions through the integrated structure of the epidermis, dermis, and hypodermis [5, 6]. The epidermis, a stratified keratinized epithelium, serves as the primary barrier against water loss and external agents [6–9]. The dermis, composed of papillary and reticular layers rich in fibroblasts, ensures mechanical support, vascularization, and tissue repair, while the hypodermis, formed by adipose and loose connective tissue, contributes to insulation, cushioning, and skin mobility [5, 7, 10].

Cutaneous wound healing involves inflammatory, proliferative, and remodeling phases regulated by coordinated interactions among inflammatory cells, fibroblasts, keratinocytes, the extracellular matrix (ECM), and microvasculature. Growth factors such as platelet-derived growth factor (PDGF), vascular endothelial growth factor (VEGF), and transforming growth factor beta (TGF-β) initiate inflammation resolution and tissue repair [10, 11], followed by re-epithelialization, collagen and elastin deposition, angiogenesis, and subsequent reorganization of collagen fibers to restore tensile strength and structural integrity [10–12].

Although histopathology remains the gold-standard for evaluating wound healing, it is invasive and time-consuming [13]. Less invasive imaging techniques, such as dermatoscopy and confocal reflectance microscopy, allow visualization of superficial structures but provide limited biochemical information [13, 14]. In this context, vibrational spectroscopic techniques, including Fourier-transform infrared spectroscopy (FT-IR) and Raman spectroscopy (RS), have gained relevance for biomedical applications by enabling biochemical and structural tissue characterization [15, 16].

RS is a non-invasive, label-free technique capable of assessing the molecular composition and spatial distribution of key skin constituents, including structural proteins (collagen, elastin and keratin), amino acids (phenylalanine, proline, hydroxyproline, tyrosine and tryptophan), lipids (phospholipids and ceramides), nucleic acids, and carbohydrates [4, 16]. It provides complementary information on the organization and functional status of the ECM [16], hydration status, barrier integrity, and tissue remodeling [4, 14], allowing characterization of spectral differences between normal and pathological conditions, such as diabetic ulcers [13, 14], and monitoring collagen regeneration during healing [4, 17].

This study aimed to perform a comparative analysis of healed skin tissue from non-diabetic (Non-Db) and diabetic (Db) Wistar rats using RS to identify diabetes related alterations in the biomolecular profile of regenerated skin tissue. It was hypothesized that diabetes modifies the relative spectral contributions of structural proteins, lipids, and amino acids during the healing process, particularly in features associated with ECM remodeling. By identifying specific spectral markers associated with skin biochemical composition, this work contributes to a better understanding of diabetic wound healing and supports the application of RS as an effective tool for evaluating normal and regenerated skin tissue.

## Materials and methods

### Ethics and animals

The experimental procedures were conducted in an accredited laboratory in accordance with the Brazilian guidelines for the care and use of laboratory animals. The study was approved by an institutional Animal Ethics Committee under an approved protocol (Protocol No. 23115.005396/2016-65).

The sample size was determined a *priori* using G*Power software (version 3.1, www.apponic.com) based on a two-sample Student *t* test (difference between two independent means). The calculation considered a two-tailed test, a significance level of 5% (*α* = 0.05), statistical power of 80% (1 − *β* = 0.80), and a large effect size (Cohen’s *d* = 1.55), resulting in a required total sample of eight animals per group (total *N* = 16). Therefore, 16 male Wistar rats were obtained from the institution’s central vivarium at 7 weeks of age, with body weights ranging from 250 to 300 g. Animals were maintained in groups of four individuals per cage in temperature-controlled rooms (22–23 °C), under a 12-h light/dark cycle, receiving standard laboratory rodent chow and water *ad libitum*.

Following acclimation, the 16 animals were randomly assigned to two primary experimental conditions: non-diabetic (Non-Db) and diabetic (Db), with an initial allocation of eight animals per group. During the experimental period, two animals were lost in each group, resulting in a final analytical sample of six animals per group.

DM was induced in the Db group through a single intraperitoneal injection of alloxan monohydrate (Sigma-Aldrich, St. Louis, MO, USA) at a dose of 150 mg/kg, prepared in sterile 0.9% saline solution and immediately administered to the animals to ensure stability and diabetogenic efficacy [18]. To prevent severe hypoglycemia resulting from the transient release of endogenous insulin, a 10% glucose solution was provided in the rats’ drinking water *ad libitum*, starting immediately after alloxan administration and maintained for 12 h. Standard rodent chow was reintroduced 1 h after DM induction. DM was confirmed 48 h after the administration of alloxan by measuring glycemia levels from the tail vein blood samples using a portable glucometer (Accu-Chek Active®, Roche Diagnostics, Indianapolis, IN, USA), and animals with blood glucose values ≥ 200 mg/dL were considered diabetic [18, 19]. The experimental procedures began at *t* = 0 (D0), after DM confirmation. At this time point (D0), baseline measurements of blood glucose and body weight were obtained for both groups. These parameters were subsequently assessed at D7 and D14 to monitor the metabolic status of the animals during the experimental period.

**Fig. 1:**
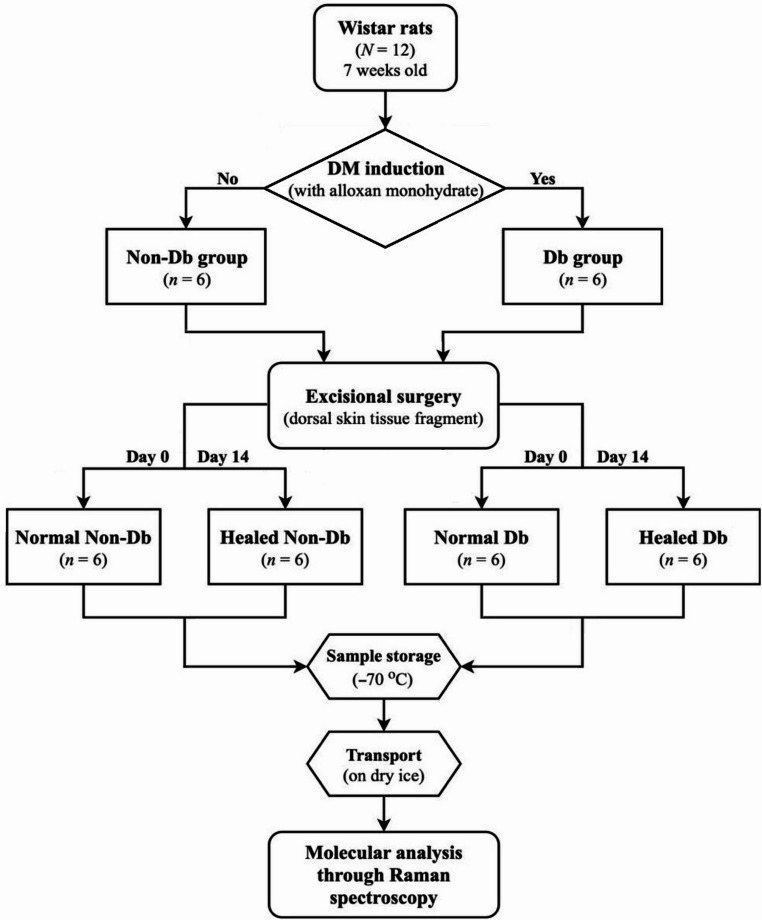
Schematic diagram of the experimental design, illustrating group allocation, DM induction, excisional skin surgery, time points for tissue collection, and biomolecular analysis through Raman spectroscopy. DM – Diabetes mellitus Non-Db – Non-Diabetic; Db – Diabetic

### Excisional skin surgery

Animals of both Non-Db and Db groups underwent excisional surgery of dorsal skin tissue in two time points as described below. To perform the excision, anaesthesia was induced through intraperitoneal administration of ketamine 10% (Quetamina®, Vetecia Laborat. Prod. Vet. Ltda., Jacareí, SP, Brazil), 80 mg/kg b.w. associated with xylazine 2% (Sedanew®, Vetecia Laborat. Prod. Vet. Ltda.), 10 mg/kg b.w. After confirmation of the anesthetic effect, the dorsal region of the rats was epilated and disinfected using chlorhexidine digluconate 0.5% (Riohex®, Rioquímica S.A., São José do Rio Preto, SP, Brazil) followed by application of ethanol 70% (Dinâmica Química Contemporânea Ltda., Indaiatuba, SP, Brazil). To standardize the size of the excision, a 3D-printed circular mould measuring 3 cm in diameter was applied to outline the site on the dorsal skin. The area was marked with a sterile dermatographic pencil (skin pencil, Alur Medical Ltda., São Paulo, SP, Brazil). The incision was done suing a sterile scalpel (No. 15 blade, Swann-Morton Ltd., Sheffield, England) and extended through the full thickness of the skin, encompassing the epidermis, dermis, and hypodermis, down to the muscular fascia. The tissue within the marked area was then subsequently excised following the mould outline using 12 cm curved Metzenbaum surgical scissor (Golgran Ind. Com. Instrum. Odontol. Ltda., São Caetano do Sul, SP, Brazil).

The skin fragments were excised at two time points. On day 0, tissue samples were collected from both Non-Db and Db groups and grouped as Normal. On day 14, tissue samples from the same groups were excised after the healing period and grouped as Healed. The excised samples were washed with 0.9% saline to remove blood residue, wrapped in aluminum foil, labeled and stored frozen at − 70 °C. Altogether, 24 samples were obtained for subsequent RS analysis (Fig. 1).

After tissue collection on day 14 day, the animals were euthanized by intraperitoneal administration of an anesthetic overdose (three times the initial dose), and the animal carcasses were collected by the institutional biological waste disposal service for incineration. 

### Raman spectroscopy

To perform the RS analysis, the frozen samples were transported in an insulated container (Termotécnica Ltda., Joinville, SC, Brazil) filled with dry ice (–78 °C). At the time of RS analysis, the collected skin tissues were passively unfrozen to room temperature. The skin samples were analyzed using a Raman spectrometer (model Dimension P-1, Lambda Solutions, Inc., Waltham, MA, USA) with a fiber-optic probe, being the laser excitation parameters as follows: 830 nm wavelength and 350 mW laser power at the probe’s distal end. The spectrometer presents a spectrograph (diffraction grating of 1200 lines/mm) coupled to a charge coupled device (CCD) camera (1340 ⋅ 100 pixels, − 75 °C), resulting in a shot noise-limited spectrum with resolution of ∼4 cm^–1^ in the spectral region from 400 to 1800 cm^–1^. The tissue samples were placed in an aluminum sample holder for laser excitation using a fiber-optic probe (Vector probe, Lambda Solutions, Inc.) positioned perpendicularly to the tissue surface, and the scattered light was collected by the probe and delivered to the spectrometer. Each point was irradiated for 3 s with 10 scans (30 s total exposure time), and six Raman spectra were collected from distinct measurement points within each tissue sample, resulting in a total dataset of 144 spectra for multivariate analysis.

After collection, the Raman spectra were pre-processed for data analysis. First, cosmic ray spikes were visually identified and removed by replacing the affected spectral point with the value of the adjacent data point, and the baseline correction was performed by fitting and subtracting a 3rd order polynomial, followed by normalization by the area under the curve (1-norm). MATLAB software (version R2007a, The MathWorks, Inc., Natick, MA, USA) was used for the baseline correction and normalization. No spectral smoothing was applied. Then, the mean normalized spectra of the groups were calculated and plotted for comparative analysis. Microsoft Excel (version 2003, Microsoft do Brasil Ltda., São Paulo, SP, Brazil) was used for mean calculation and plotting. Spectra were then submitted to exploratory analysis by principal component analysis (PCA) to investigate spectral differences and biomolecular variations among the experimental groups, using the individual spectra without prior averaging. 

### Exploratory analysis

PCA was applied as an exploratory multivariate technique to reduce the dimensionality of the Raman dataset and to characterize the biomolecular composition of skin tissue, highlighting biochemical variations among samples. In PCA, the dataset is decomposed into scores and loadings based on the covariance matrix [20]. Loading plots provide information on the distribution and clustering of samples in the principal component space, whereas score plots reveal the spectral variables responsible for the observed variance and sample discrimination. The loadings correspond to the coefficients of the principal components and indicate the contribution of each original spectral variable to each component through positive or negative values. The latent variables quantified the proportion of total variance explained by each principal component, with decreasing variance for successive components. PCA was performed in MATLAB software (princomp.m function) using the full spectral range (400–1800 cm^–1^) without selection or exclusion of specific spectral regions. The PCA variables were described according to MATLAB’s nomenclature as loadings (coefficients), scores, and latent variables. A cumulative variance threshold of 97% was adopted as the criterion for retaining the principal components for interpretation.

Statistical analysis was conducted using OriginLab software (version 2025, OriginLab Corporation, Northampton, MA, USA). The null hypothesis (H_0_) assumed no differences among groups, whereas the alternative hypothesis (H_1_) assumed significant differences among groups. A significance level of 5% (*p* < 0.05) was adopted. Data normality was assessed using the Kolmogorov-Smirnov test. Normally distributed data were analyzed using one-way analysis of variance (ANOVA) followed by the Tukey-Kramer post-hoc test, whereas non-normally distributed data were analyzed using Kruskal-Wallis test (non-parametric ANOVA) followed by Dunn post-hoc comparisons. Statistical comparisons were performed using the individual spectra acquired from distinct measurement points within each tissue sample.

## Results and discussion

### DM confirmation and skin healing macroscopic assessment 

DM was confirmed in the Db group 48 h after alloxan administration based on blood glucose values ≥ 200 mg/dL. At baseline (D0), body weight was similar between groups (285.2 ± 18.9 g in Non-Db vs. 278.2 ± 20.3 g in Db), while blood glucose levels were markedly higher in the Db rats (378.5 ± 25.4 mg/dL) compared to Non-Db ones (99.2 ± 6.0 mg/dL). Comprehensive body weight and blood glucose values for all experimental time points are provided in Table 1.

**Table 1:** Body weight and blood glucose values of Wistar rats in Non-Db and Db groups across experimental time points (D0, D7, and D14)

Time point	Non-Db		Db	
	Weight (g)	Glucose (mg/dL)	Weight (g)	Glucose (mg/dL)
D0	285.2 ± 18.9	99.2 ± 6.0	278.2 ± 20.3	378.5 ± 25.4
D7	288.2 ± 19.4	99.5 ± 3.1	248.3 ± 22.2	402.5 ± 39.9
D14	299.8 ± 12.2	100.3 ± 6.2	230.7 ± 14.5	397.0 ± 13.8

**Fig. 2:**
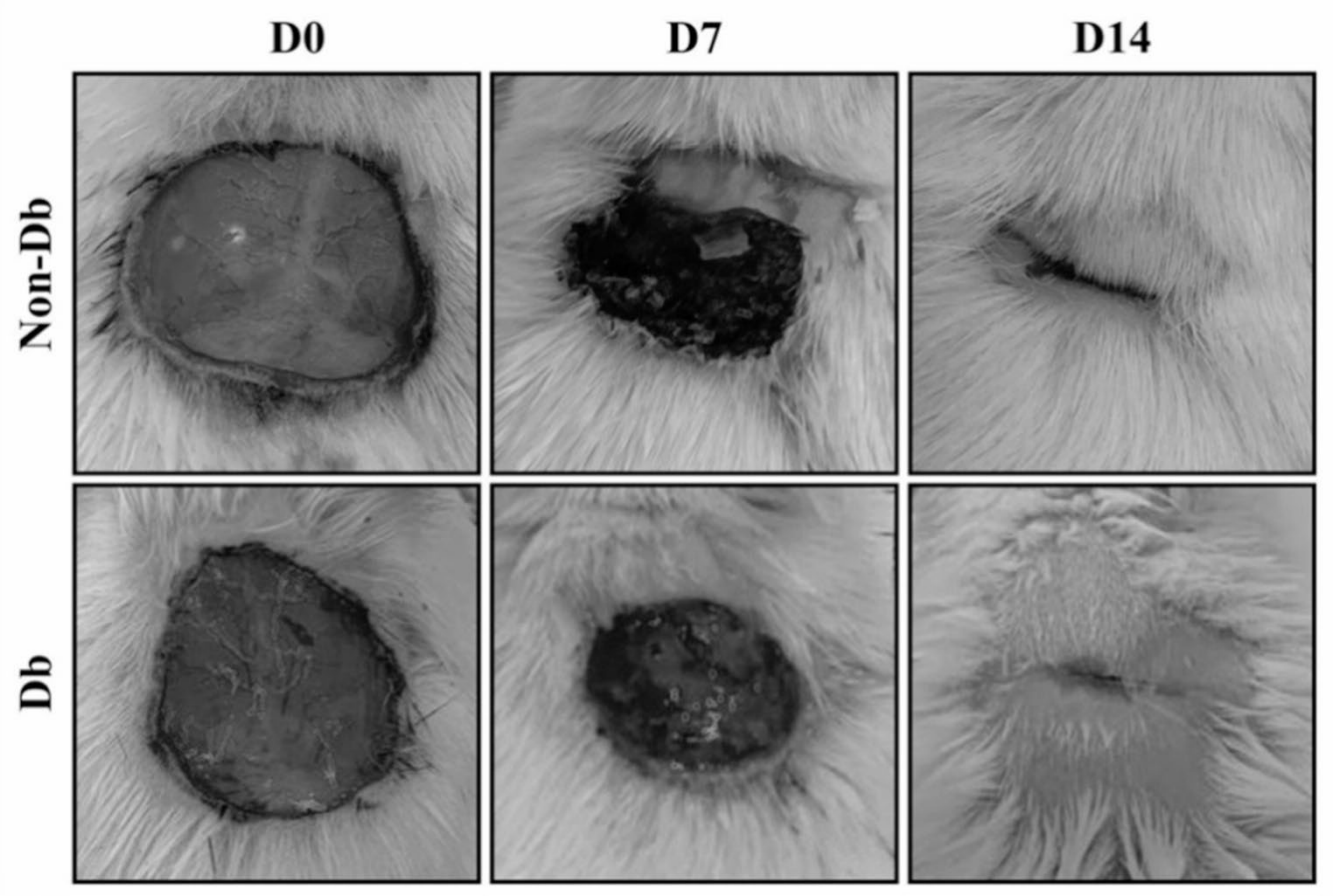
Macroscopic evaluation of the skin healing process. Representative images illustrating the initial full-thickness excision at day 0 (D0) and the subsequent progression of wound closure at day 7 (D7) and day 14 (D14) in the Non-Db and Db groups

Macroscopic assessment was performed to monitor the progression of skin healing in both groups (Fig. 2). On day 0 (D0), immediately following the full-thickness rat skin excision (epidermis, dermis, and hypodermis), the surgical wounds exhibited a well-defined circular area, delineated by the 3-cm diameter mold detailed in the Methods section. By D7, hallmarks of the proliferative phase were evident, including wound contraction, formation of a fibrinous crust, and initial tissue coverage. By D14, the lesions displayed an advanced stage of repair, characterized by near-complete closure, re-epithelialization, and ongoing remodeling. These macroscopic observations validated the selection of D0 (Normal) and D14 (Healed) as representative time points for Raman spectral analysis.

### Raman spectra 

**Fig. 3:**
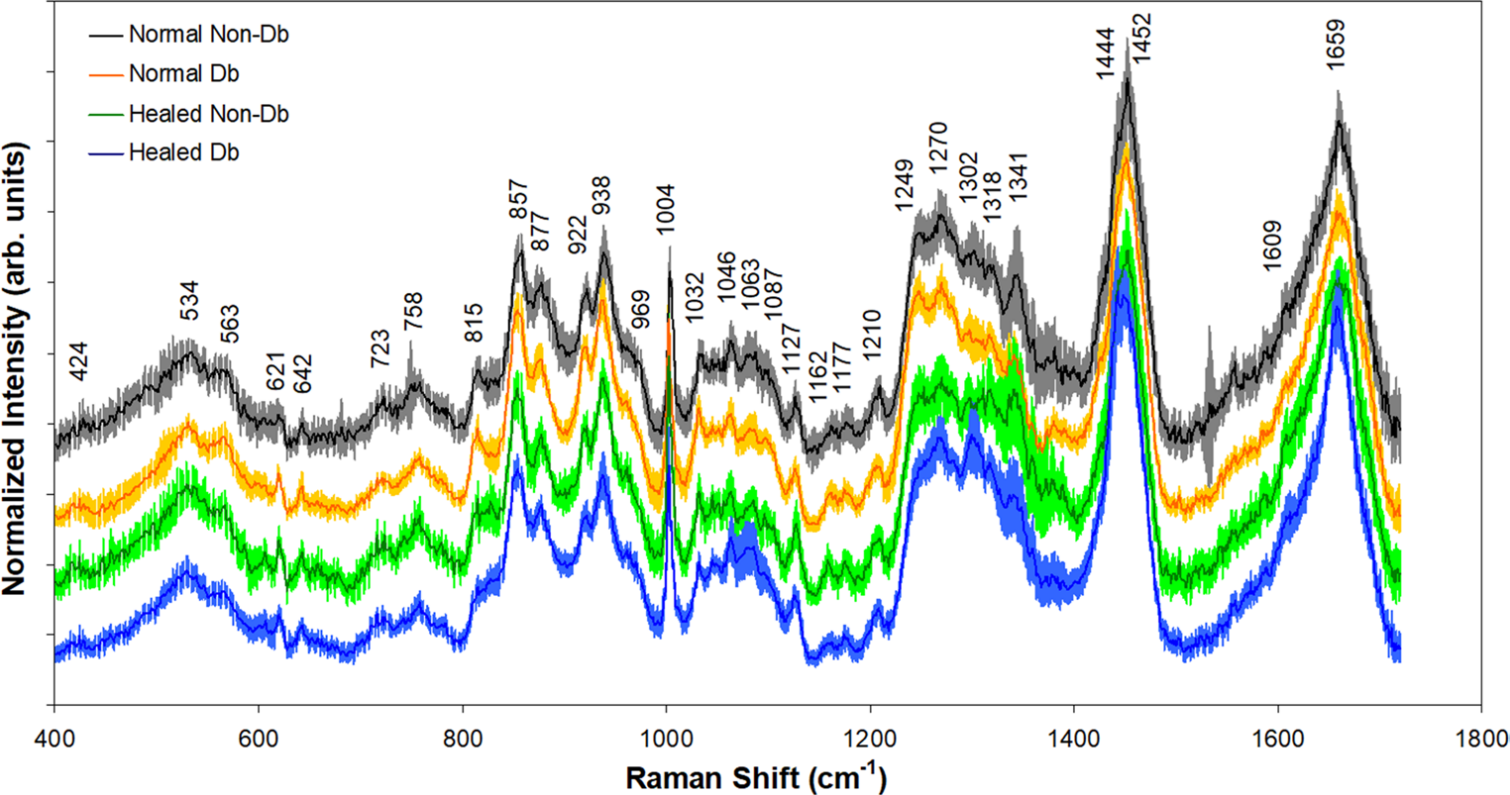
Mean raman spectra of wistar rat’s skin tissues from Non-Diabetic (Non-Db) and Diabetic (Db) groups at two-time intervals: *t* = 0 (Normal group) and *t* = 14 days, (Healed group). Standard deviation (SD) shadows were also presented

Figure 3 presents the Raman spectra of Wistar rat’s skin tissue of the groups Non-Db and Db at the time intervals *t* = 0 (Normal) and *t* = 14 days (Healed). The spectral features were assigned to the main biomolecular components of rat’s skin tissue: structural proteins and lipids. Online Resource 1 (Supplementary Table 1) lists the corresponding Raman band positions and vibrational assignments reported in previous literature [21–31]. The assignments highlight the main biochemical components of the skin in both dermis and epidermis: structural proteins such as collagens (rich in glycine, proline, and hydroxyproline), elastin (rich in alanine and lysine) and keratin (rich in amino-acids with S–S bonds), as well as amino-acids with aromatic ring (phenylalanine, tryptophan, and tyrosine) [3, 22–26, 32, 33], lipid components that constitute cellular membranes and tissue structures, including the stratum corneum (ceramides and cholesterol), stratum granulosum (phospholipids and sphingomyelin), basal layer (cellular phospholipids), sebaceous glands (mainly triglycerides) and hair follicles (follicular ceramides and sebum) [3, 23–26, 33]. Features from nucleic acids, primarily from the cell nucleus (DNA/RNA markers) [4, 24, 34] and from cellular components, such as cytochromes located in mitochondria [35], may also contribute to the spectrum by overlapping with specific protein/amino-acid and lipid bands. Protein components may be divided into structural proteins and free amino acids, while lipid components may be categorized according to whether they contain saturated (–CH_2_) or unsaturated (C=C, =C–H) bonds, with some peaks serving as markers of phospholipids (most notably the choline headgroup peak at 723 cm^–1^).

As pointed out, protein-related features prevailed in the mean Raman spectra of all groups, where the bands with peaks at 856, 877, 922, 938, 1004, 1249, 1271, 1341, 1452 and 1659 cm^–1^ were attributed to structural proteins such as collagen I/III, elastin, and keratin. Lipids, phospholipids and ceramides were observed at the peaks at 723/758, 1073/1087, 1127, 1302, 1444 and 1659 cm^–1^. The sharp peak at 1004 cm^–1^ is assigned to aromatic ring breathing of phenylalanine and tryptophan, and together with the peak at 1452 cm^–1^ (CH_2_/CH_3_ modes) well characterizes the protein-nature of the spectrum. The peaks at 1270 and 1341 cm^–1^ can be assigned to amide I and amide III in structural proteins (collagen I/III, elastin and keratin). The broad band at ∼1659 cm^–1^ has contributions from amide I band (C=O stretching) of proteins and C=C band from unsaturated lipids.

Visual inspection of Fig. 3 showed that the most intense Raman peaks overlapped and exhibited comparable relative (normalized) intensities among groups, indicating a similar overall biochemical composition in samples from both Non-Db and Db groups, independently on the time of healing. Consequently, no significant biochemical differences were identified at first evaluation. Therefore, multivariate analysis was applied to the Raman data to clarify these spectral differences, as described in the following sub-section. 

### Multivariate analysis by PCA

**Fig. 4:**
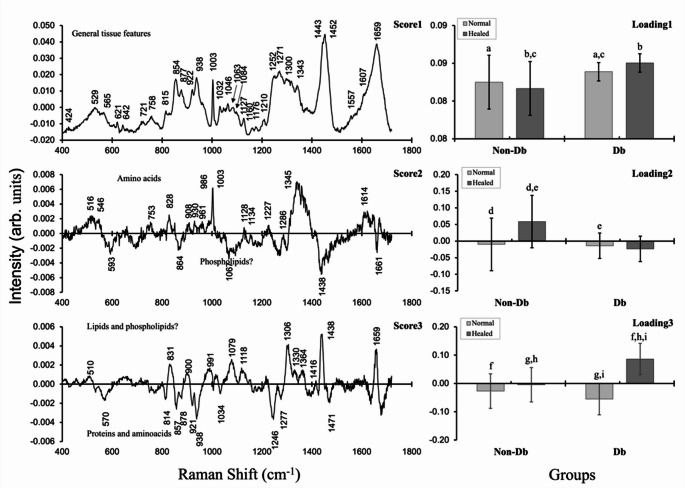
Principal component variables (score and loadings) from Raman spectra of Wistar rat’s skin tissue (Score1-Score3, left; Loading1-Loading3, right). The Scores identified the Raman bands that contributed most to the biochemical variance within the tissue, while the loadings (bar plots) showed differences in spectral intensities between Normal (*t* = 0) and Healed (*t* = 14 days) tissue samples in the Non-Db and Db groups. The letters in parentheses above the error bars in the loadings indicated statistically significant differences between tissue conditions (Normal and Healed) across groups (Non-Db and Db), as determined by one-way ANOVA or Kruskal-Wallis tests (*p* < 0.05). Groups sharing the same letter present statistically significant differences from each other. Non-Db, non-diabetic; Db, diabetic

PCA was applied to the Raman spectra, and the results are presented in Fig. 4. Based on the decomposition of the covariance matrix, PCA identified the spectral features of the skin tissue that most contributed to the biochemical variability among the experimental groups while reducing the dimensionality of the dataset. This approach enabled the identification of relevant vibrational features responsible for group differentiation. The first six principal component explained 94.2% (Loading1), 1.85% (Loading2), 0.79% (Loading3), 0.34 (Loading4), 0.24 (Loading5), and 0.16% (Loading6) of the total variance, resulting in a cumulative variance of 97.6%. In this study, the first three PCA variables (Score1/Loading1 to Score3/Loading3), accounting for 96.85% of the total variance, were presented in Fig. 4, and the PCA variables 4 to 6 were presented in Online Resource 2.

PCA revealed significant differences in the biochemical signatures of skin tissue among the experimental groups. In Fig. 4, the spectral signatures seen in the Scores are in the same positions of the Raman bands that contributed most to biochemical variability of skin tissue. The spectra assignments of the features identified in Score1 to Score6 and their associated with skin biochemical constituents were described in Online Resource 1. The bar plots of the loading intensities between Normal and Healed samples in the Db and Non-Db groups reflect differences in the relative spectral contributions of these biomolecular constituents among groups. In an overall analysis, the Db group showed distinct vibrational patterns compared to the Non-Db group, indicating diabetes-related alterations in the molecular composition of both Normal and Healed skin tissue. The need for a high number of PCA variables to explain the spectral variability indicated that the biochemical changes during the healing process are complex.

The comparison of the loading’s intensities among the experimental groups at the time intervals for Normal (*t* = 0) and Healed (*t* = 14 days) (Fig. 4) resulted in statistically significant differences for Loading1 (Kruskal-Wallis, *p* = 0.00001), Loading2 (Kruskal-Wallis, *p* = 0.00001) and Loading3 (ANOVA, *p* = 0.00001). These findings indicate that the spectral variability captured by the first three principal components differed among the Normal and Healed tissues from the Non-Db and Db groups. The biochemical interpretation of the Raman features identified in the PCA variables was based on Raman band assignments reported in the literature for biological tissues and fluids and should therefore be considered a spectroscopic interpretation rather than direct biochemical confirmation of specific molecular constituents.

Score1 presented spectral features like the mean spectrum in Fig. 4. Briefly, the peaks are assigned mostly to structural proteins, representing dermal and epidermal structures responsible for strength and elasticity, and lipids in less extent, representing components of the skin barrier, essential for maintaining tissue integrity, hydration and protective function [26, 36]. Loading1 showed higher intensity for the groups Db compared to Non-Db, even higher for the Healed Db group, even though not all the comparisons were statistically significant. These changes suggest differences in proteins-related spectral patterns between diabetic and non-diabetic skin tissues. Such differences may be associated with ECM remodeling during wound healing. The high intensity in the Db groups, particularly in the Healed Db group, is consistent with altered ECM remodeling in diabetic wound healing [37–39]. The presence of lipid-related vibrational bands in Score1 suggests partial preservation of the biochemical barrier of the skin’s stratum corneum [6, 39]. The high variability (high error bars) in the Non-Db groups indicate greater biochemical heterogeneity within these groups.

Score2/Loading2 presented positive features that could be assigned to proteins and represent dermal and epidermal structures (collagen, elastin and keratin), which contribute to tissue strength and organization [12, 16, 17, 22–26, 32, 33, 36, 40], and were significantly higher for the Healed Non-Db group compared to the Normal Non-Db group. Negative features in Score2/Loading2 could be assigned to lipids, which were (not significantly) higher for the Healed Db group, corroborating the findings of high lipid content in the skin of the diabetic rats. The predominance of features related to structural proteins in the Healed Non-Db skin tissue suggests differences in ECM remodeling compared with the Normal Non-Db groups, consistent with distinct protein-driven remodeling processes during skin repair in non-diabetic rats. The presence of lipid features in the Healed Db group indicates difference in epidermal lipid components compared with the skin from non-diabetic rats.

Score3/Loading3 showed distinct features: positive peaks assigned to lipids (indubitable peaks of lipids at 1079, 1306, 1438 and 1659 cm^–1^) [23–26, 36, 40] and negative peaks assigned to structural proteins (indubitable peaks of collagen at 857, 878, 921, 938 and 1246 cm^–1^) [17, 22–26, 32, 33, 36, 40]. Structural proteins such as collagen, elastin and keratin, contribute to dermal tensile strength, dermal organization and fibrillar architecture [5, 12, 25]. Lipid-related compounds such as triolein (fatty acids), ceramides, and phospholipids in some extent, are constituents of the stratum corneum lipid barrier, which are essentials for maintaining cutaneous integrity [5, 9]. Both Normal Non-Db and Db groups suggested protein-rich tissue architecture, which is expected for a normal skin. The stronger protein-related spectral contribution observed in the Db group suggests differences in collagen content due to fibroblast activity and tissue organization. In contrast, the Healed Db group showed a lipid-rich spectral profile, which may reflect increased contributions from cellular membrane components associated with infiltrating inflammatory cells, fibroblasts, and differentiating keratinocytes. As re-epithelialization progresses, the progressive reconstruction of the epidermal lipid barrier characterizes a renewed production of ceramides, cholesterol, and free fatty acids by differentiating keratinocytes [17, 41–44]. Consequently, Raman bands associated with lipids may represent either membrane-rich cellular turnover or the restoration of the stratum corneum’s barrier lipids. The Non-Db groups showed lower amounts of these biomolecules, consistent with the preserved integrity and ordered arrangement of normal layers in skin of rats without diabetes, even in injured tissue.

The spectral profile of non-diabetic normal skin was primarily determined by dermal and epidermal proteins, extracellular matrix components and complementary lipid contribution. In brief, the Raman spectra of skin were dominated by structural proteins with a complementary lipid contribution, reflecting the biochemical organization of rat skin tissue. Protein-related bands (including proline/hydroxyproline and amide bands at 856, 877, 922, 938, 1004, 1249, 1271, 1341, 1452, and 1659 cm⁻¹) were mainly with spectral features commonly attributed to types I and III collagen from the extracellular matrix, which reflects the collagen organization and structural integrity of skin [17, 27, 45]. Additional contributions from elastin and epidermal keratins were consistent with bands related to protein secondary structure and CH_3_/CH_2_ modes (1318, 1341, 1427, and 1452 cm⁻¹), characterizing the epidermis and the epidermal-dermal interface [28]. Lipid features reflect the presence of phospholipids (723, 758, and 1087 cm⁻¹) and structural ceramides (1063, 1127, and 1302 cm⁻¹) related to cell membranes and skin barrier function, in agreement with Raman studies of intact rodent [28, 41] and humans [44] skin and wound healing models [28, 41].

In the non-diabetic tissue, the normal skin showed a higher protein contribution than healed skin. The spectral pattern seen in Score1/Loading1 and Score3/Loading3 was associated with a matrix enriched with type I fibrillar collagen, supported by proline (854–859 and 922 cm⁻¹), hydroxyproline (877–886 and 938 cm⁻¹), and amide I/III (1210–1271, 1607, and 1659 cm⁻¹) bands, corresponding to a more organized extracellular matrix [27, 45] in the normal skin. In turn, the healed skin exhibited reduced protein signatures and a redistribution of collagen features, consistent with remodeling processes and a higher contribution of type III collagen, typical of granulation tissue and intermediate healing stages [24, 28, 41]. Amino-acid related bands differed between groups, with increased contributions in healed and normal skins. In the healed skin, Score2/Loading2 was characterized by phenylalanine and bands of aromatic amino-acids in protein (743, 753, 1003. 1340, 1350, 1358, and 1614 cm^–1^). Overall, these findings suggest differences in dermal structural protein content and organization between healed and normal tissues [17, 25, 27, 45]. The lipid contribution was lower in normal tissue, seen by the reduced bands related to phospholipids seen in Score2/Loading2 (peaks at 593, 864, 1067, 1272, 1438, and 1661 cm^–1^), consistent with preserved lipid barrier organization. In Healed Db (Score3/Loading3), this lipid phospholipid signature was dominated by bands at 1079, 1118, 1306, 1330, 1364, 1416, 1438, and 1659 cm⁻¹. In contrast, the increased lipid phospholipid contribution observed in Healed Db (Score3/Loading3) suggested membrane reorganization associated with tissue repair. Compared with Score2/Loading2, Score3/Loading3 revealed a more specific and stronger phospholipid signature in the Healed Db group, suggesting increased membrane remodeling and phospholipid turnover during tissue repair under diabetic conditions.

In the diabetic tissue, spectral features due to collagen (types I and III), amino acids, and membrane lipids showed prominent contributions to the Raman profiles and were consistent with molecular patterns reported for diabetic wound repair [34, 39]. In Healed Db group, spectral features associated with collagen and structural protein remodeling were supported by proline (854 cm^–1^), hydroxyproline (877 and 938 cm^–1^), amide III (1210, 1252, and 1271 cm^–1^), and amide I (1607 and 1659 cm^–1^) bands (Score1/Loading1), with additional collagen/protein contributions (922, 1343, 1443, 1452, and 1557 cm^–1^), suggesting a less mature collagen organization, which is a characteristic of ongoing remodeling and consistent with delayed collagen maturation in diabetic wounds [17, 21, 34, 45]. Protein related differences in diabetic tissue were further supported in Normal Db (Score3/Loading3) by aromatic amino acid and protein structural bands (570, 814, 857, 878, 921, 938, 1034, 1246, 1277, and 1471 cm^–1^) and by additional protein contributions in Score4/Loading4 (930, 1002, 1028, 1232, 1334, and 1420 cm^–1^), reinforcing altered protein organization and collagen turnover under diabetic conditions [17, 42, 43].

RS enabled the molecular characterization of Wistar rat skin by capturing vibrational signatures of proteins, lipids, and amino-acids associated with tissue organization of both normal and healed tissue. The technique identified protein profiles dominated by collagen, lipid contributions related to membrane and barrier properties, and amino-acids features linked to ECM architecture, as reported in Raman studies of cutaneous tissue and during healing [13, 16, 24, 28]. These findings support the application of RS as a non destructive, label free approach for monitoring spectral changes associated with extracellular matrix remodeling and epidermal barrier reorganization during physiological skin healing [29, 36], and further highlight the potential of RS associated with PCA as a tool for monitoring biochemical changes during diabetic wound healing. Collectively, the results indicate that further studies under more complex experimental conditions, including in vivo analyses, independent experimental cohorts, and external validations procedures, are required to validate these findings, assess the reproducibility of the PCA results, and strengthen their applicability in broader biological contexts and translational relevance to human tissue. In addition, the relatively small number of animals and the use of a single experimental cohort should be considered when interpreting the present findings. Although the sample size was determined through an a priori power analysis, studies involving larger independent cohorts would provide further confirmation of the reproducibility of the observed spectral patterns.

## Conclusion

The Raman spectra of Wistar rat skin were mainly characterized by bands attributed to structural proteins (collagen types I/III, elastin, and keratin), with relevant contributions from amino acid related bands, particularly the ones with aromatic rings (phenylalanine, tryptophan, and tyrosine), and complementary lipid signatures associated with membrane and barrier organization, including ceramides and phospholipids. Although the mean Raman spectra appeared visually similar across groups, PCA identified remodeling-related spectral signatures that differentiated the experimental conditions.

Normal Non-Db tissue showed a profile dominated by dermal and epidermal structural proteins, consistent with intact skin organization. After healing, Healed Non-Db tissue preserved the main protein related features but exhibited increased ECM-associated contributions, suggesting repair primarily driven by structural protein remodeling and collagen redistribution.

Under diabetic conditions, Normal Db tissue displayed a distinct biochemical signature compared with Normal Non-Db, with a relatively stronger amino acid related contribution, suggesting baseline diabetes-associated alterations in skin biomolecular organization. Healed Db tissue exhibited a distinct spectral profile characterized by altered protein related features consistent with collagen remodeling, followed by increased lipid related signatures, particularly membrane phospholipids. Overall, diabetes mellitus was associated with a distinct repair pattern characterized by modified collagen-related features and enhanced membrane lipid signatures in healed diabetic skin.

These findings indicate that Raman spectroscopy combined with PCA can detect spectral patterns associated with biochemical alterations during diabetic wound healing. However, additional investigations using more complex and in vivo experimental models are required to confirm these results and to establish their translational relevance across different biological systems, including human tissue.

## Supplementary Information

Supplementary Material 1.

Supplementary Material 2.

## Data Availability

The data supporting the findings of this study are available from the corresponding authors upon reasonable request.
