# Supplementary material for: Comparative biomolecular analysis of normal and healed skin tissue from diabetic and non-diabetic rats using Raman spectroscopy
Source: Lasers Med Sci. 2026 Jul 25;41(1):165. doi: 10.1007/s10103-026-04961-x (PMC13401533; doi:10.1007/s10103-026-04961-x)
Supplement: Supplementary file 1 — Supplementary Material 1. [file 10103_2026_4961_MOESM1_ESM.pdf]

Online Resource 1. Positions of the most intense Raman peaks observed in the mean spectra of the non-diabetic Wistar rat's skin at the time interval $t = 0$ (Normal Non-Db group, **Fig. 2**) and in the loadings (PCs) from the PCA (**Fig. 3**) with the corresponding vibrational assignments of these peaks based on the recent literature.

Biomolecule class*	Mean spectrum (cm^{-1})	PC loading (cm^{-1})	Suggested vibrational assignment	Main biochemical component	References
Proteins and amino acids	534	529 (PC1) 516, 546 (PC2), 510 (PC3, PC6), 508 (PC5)	S–S stretching (disulfide bridge, gauche conformation)	Proteins (keratin)	[22-24]
	563	565 (PC1), 570 (PC3)	S–S stretching (disulfide bridge, *trans* conformation)	Proteins (keratin)	[22]
	621	621 (PC1), 582 (PC6)	Ring *breathing*	Proteins (hydroxyproline/phenylalanine)	[23,30]
	642	642 (PC1), 639 (PC4), 617 (PC5), 604, 665 (PC6)	C–C ring vibration – side chain	Proteins [proline(?)/tyrosine/cysteine]	[23,30]
	758	758 (PC1), 743, 753 (PC2), 749 (PC4, PC5)	Indole ring breathing	Proteins (tryptophan)	[23,25,29]
	857	815, 854 (PC1), 828 (PC2), 814,	C–C stretching – ring	Proteins (collagen – proline/hydroxyproline, keratin)	[21,24-27,29]

Biomolecule class*	Mean spectrum (cm^{-1})	PC loading (cm^{-1})	Suggested vibrational assignment	Main biochemical component	References
		831, 857 (PC3), 859 (PC4), 856 (PC5)			
	877	877 (PC1), 908 (PC2), 878, 900 (PC3)	C–C stretching	Collagen [glycine/hydroxyproline/tryptophan(?)]	[21,23,25,26]
	922	922 (PC1), 908 (PC2), 921 (PC3)	C–C ring vibration – side chain	Collagen (proline), keratin	[21,23,26]
	938	938, (PC1, PC3), 930, 986 (PC2), 991 (PC3), 930 (PC4), 937 (PC6)	C–C stretching (backbone skeletal chain)	Collagen (proline/hydroxyproline)	[21,26,27,29]
	1004	1003 (PC1, PC2, PC6), 1002 (PC4), 1006 (PC5)	Aromatic ring breathing	Proteins (phenylalanine/tryptophan, keratin)	[21,26,29,30]
	1032	1032, 1046 (PC1), 1034 (PC3), 1028 (PC4)	In-plane C–H stretching – aromatic ring	Proteins [phenylalanine/proline(?)]	[21,30]

Biomolecule class*	Mean spectrum (cm^{-1})	PC loading (cm^{-1})	Suggested vibrational assignment	Main biochemical component	References
	1100	1134 (PC2)	(?)	Protein [elastin/collagen, keratin(?)]	[26]
	1177	1176 (PC1), 1173 (PC3)	Vibration (side-chain)	Proteins (hydroxyproline/tyrosine)	[21,25,26]
	1210	1210 (PC1)	C–C stretching – side chain coupled to aromatic ring	Proteins (phenylalanine)	[21,26,30]
	1249	1252 (PC1), 1227 (PC2), 1246 (PC3), 1232 (PC4), 1230 (PC6)	Amide III (*random coil*/β-*sheet*)	Proteins	[21,23,25,29]
	1271	1271, 1300 (PC1), 1286 (PC2), 1277 (PC3), 1268 (PC5), 1298 (PC6)	Amide III (α-*helix*)	Proteins	[21,23,26,29]
	1318	1340 (PC2), 1330 (PC3), 1234 (PC4), 1345 (PC4, PC5)	C–H deformation (*rocking*)	Structural proteins (collagen I/collagen III/elastin), glycine	[21,26,27]
	1341	1343 (PC1), 1350 (PC2)	CH_2 deformation (*wagging*); ring vibration (proline)	Structural proteins (collagen III/elastin), proline, tryptophan, glycine	[21,26,27,29]

Biomolecule class*	Mean spectrum (cm^{-1})	PC loading (cm^{-1})	Suggested vibrational assignment	Main biochemical component	References
	1427	1412 (PC2)	CH_2 (aliphatic side chain) & CH_3 deformations	Overlap between lipid and protein signals (including tryptophan)	[23]
	1452	1452 (PC1)	CH_2 deformation – *scissoring* & CH_3 *–bending*	Proteins (aliphatic side chains); keratin	[21,29]
	1557	1557 (PC1)	Amide II / indole ring vibrations	Proteins (tryptophan)	[27,29]
	1609	1607 (PC1)	C=C stretching (aromatic ring)	Collagen (phenylalanine)	[27,29,30]
	1659	1659 (PC1, PC3, PC4), 1614 (PC2), 1648 (PC6)	C=O stretching of peptide backbone – amide I (α*-helix*)	Proteins	[21,27,29]
Lipids, phospholipids and cholesterol	723	721 (PC1), 734 (PC6)	Symmetric $N+(CH_3)_3$ stretching – choline; steroid ring breathing – cholesterol	Phospholipids (choline headgroup); cholesterol (stratum corneum)	[23,28]
	1063	1063 (PC1), 1067 (PC2)	C–C stretching (*trans*)	Saturated lipids (including ceramide)	[21,25,29,31]
	1087	1084 (PC1), 1079 (PC3)	C–C stretching – acyl segment (trans conformation); P–O stretching	Unsaturated lipids; phospholipids	[25,29,31]
	1127	1127 (PC1), 1128 (PC2), 1118 (PC3), 1144 (PC5), 1134 (PC6)	C–C stretching	Saturated lipids (including ceramide)	[21,23,24,28-31]

Biomolecule class*	Mean spectrum (cm^{-1})	PC loading (cm^{-1})	Suggested vibrational assignment	Main biochemical component	References
	1271	1172 (PC2)	=CH deformation	Unsaturated lipids (appears together with the band at 1659 cm^{-1})	[23,26,29,31]
	1302	1317, 1272 (PC2), 1306 (PC3), 1420 (PC4)	CH_2 deformation (*twisting*/*wagging*)	Lipids (including ceramide) – narrow peak in saturated lipids (due to greater chain order)	[23,24,29,31]
	1444	1443, 1452 (PC1), 1438 (PC2), 1364, 1416, 1438 (PC3), 1452 (PC4), 1389, 1450 (PC5)	CH_2/CH_3 deformation	Lipids (including ceramide); cholesterol (stratum corneum)	[21,23,24,29,31]
	1659	1659 (PC1, PC3, PC4), 1471 (PC3)	C=C stretching; cholesterol vibration	Unsaturated lipids (appears with the band at 1271 cm^{-1}); cholesterol (stratum corneum)	[23,24,29,31]
Nucleic acids	~550	593 (PC2)	(?)	RNA (overlapped with proteins/keratin)	[23]
	723	721 (PC1)	(?)	DNA/RNA (overlapped with proteins/lipids/phospholipids)	[23]
	~780	—	Pyrimidine ring breathing; PO_2^- stretching (very weak in the spectrum)	DNA/RNA (nucleic acid marker)	[23,25,26,29]

Biomolecule class*	Mean spectrum (cm^{-1})	PC loading (cm^{-1})	Suggested vibrational assignment	Main biochemical component	References
	~1230	1230 (PC6)	Asymmetric PO_2^- stretching (backbone) (very weak in the spectrum)	DNA/RNA (overlapped with proteins/lipids)	[29]
	~1325	1358 (PC2)	Ring vibration (purine)	DNA/RNA (overlapped with structural proteins/amino acids)	[23,29]
Other cellular components (mitochondria)	756	758 (PC1)	Pyrrole ring breathing	Cytochrome C	[26,29]
	1127	1127 (PC1)	C–N stretching; pyrrole ring breathing – heme	Cytochrome C	[29,30]
	1302	1300 (PC1)	C–H deformation – heme	Cytochrome C	[26,29]
	~1580	—	Pyrrole ring breathing (C=C) – heme	Cytochrome C (overlapped with structural proteins/amino acids)	[29]

Amide I – C=O stretching and C–N stretching (to a lesser extent)

Amide II – C–N stretching, N–H deformation, and C=O stretching (to a lesser extent)

Amide III – C–N stretching and N–H deformation
