# Supplementary material for: Comparative biomolecular analysis of normal and healed skin tissue from diabetic and non-diabetic rats using Raman spectroscopy
Source: Lasers Med Sci. 2026 Jul 25;41(1):165. doi: 10.1007/s10103-026-04961-x (PMC13401533; doi:10.1007/s10103-026-04961-x)
Supplement: Supplementary file 2 — Supplementary Material 2. [file 10103_2026_4961_MOESM2_ESM.pdf]

Online Resource 2. PCA components 4–6: score plots, loadings, and spectral interpretation.

Plot of the PCA components 4 to 6.

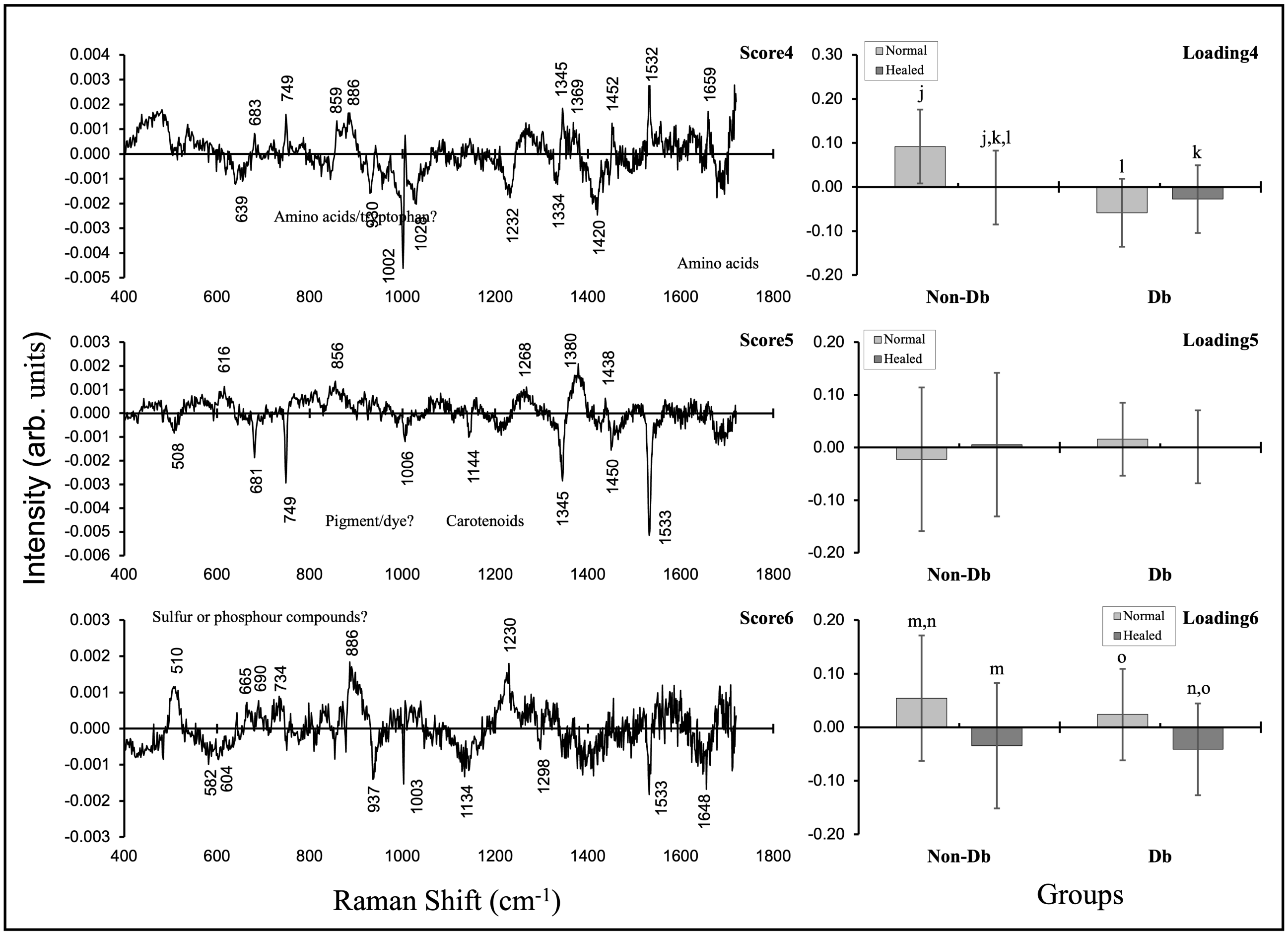

Results of the PCA scores and loadings.

Score4/Loading4 showed positive features that can be assigned to remnant protein features seen in Score3 (the peaks at 859 and 886 cm^{-1} confirm the assignments to collagen III), which were higher for the Normal Non-Db group (as was with moderate intense for Loading3). The negative features can be assigned to amino acids (tryptophan/tyrosine), which were higher for both Normal and Healed Db groups, even with higher intensity for the Normal Db group. The presence of amino-acid features in the groups with diabetes highlight distinct molecular remodeling between normal and regenerated skin under diabetic and non-diabetic conditions.

Score5/Loading5 showed negative peaks (508, 681, 749, 1006, 1144, 1345, 1450 and 1533 cm^{-1}) which corresponded primarily to amino-acid vibrations. Bands in the 749–1006 cm^{-1} region are consistent with tryptophan and phenylalanine ring modes, while features near 1345–1533 cm^{-1} have

been associated with mixed CH_3/CH_2 deformation modes and aromatic amino-acid contributions described in Raman studies of skin and wound healing tissue [17,41,45]. These spectral characteristics did not differ significantly between groups and therefore reflected individual biochemical variability rather than group-defining differences linked to diabetes.

Score6/Loading6 presented positive bands associated with structural proteins (510, 886 and 1230 cm^{-1} from keratin and collagen III) and negative bands also assigned to proteins and remnant bands of amino acids. The groups Normal showed the structural proteins in higher amount, and the groups Healed showed the proteins and amino acids in higher amount. As noted before for other Scores/Loadings that showed positive features of proteins and negative features of proteins and amino acids, these apparent distinct and conflicting condition in fact reflect the complexity of the molecular processes involved in tissue repair under diabetic conditions compared to the non-diabetic cases, where the protein synthesis and turnover during the healing process are remodeled by the presence of diabetes.
